# Knowledge Translation Initiative to Improve Interdisciplinary Approaches to Psychosocial Oncology Among Community Stakeholders in Rural Regions of British Columbia

**DOI:** 10.3390/ijerph22121789

**Published:** 2025-11-26

**Authors:** Melba Sheila D’Souza, Louise Racine, Ruby Gidda, Prashant Kumar Pradhan, Arsh Sharma, Karma Lalli, Ashwin Nairy, Alice Sheethal Rasquinha

**Affiliations:** 1 School of Nursing and Population Health, Thompson Rivers University, Kamloops, BC V2C 0C8, Canada; 2 College of Nursing, University of Saskatchewan, Saskatoon, SK S7N 5E5, Canada; louise.racine@usask.ca; 3 School of Nursing, University of British Columbia, Vancouver, BC V2S 0C2, Canada; ruby.gidda@bccancer.bc.ca; 4 Clinical Capacity Optimization, BC Cancer, Abbotsford, BC V3G 2R9, Canada; 5 Primary Care Transformation Data Planning and Initiatives, Interior Health Authority, Kelowna, BC V1Y 6V8, Canada; prashant.pradhan@interiorhealth.ca; 6 Department of Microbiology and Immunology, Faculty of Science, University of British Columbia, Vancouver, BC V6T 1Z4, Canada; asharm.lab@gmail.com (A.S.); anairy@student.ubc.ca (A.N.); 7 Emergency Department, Nicola Valley Hospital, Merritt, BC V1K 1H6, Canada; karma.lalli@interiorhealth.ca; 8 Faculty of Science, Bob Wright Centre, University of Victoria, Victoria, BC V8P 5C2, Canada; alicerasquinha@uvic.ca

**Keywords:** community-based participatory action, implementation science, oncology, patient engagement, health systems, community nursing, knowledge translation, knowledge mobilization, rural health, remote communities

## Abstract

Background: This study reports on a community engagement knowledge-translation world café hosted in British Columbia, built on the research project “Enhancing cancer navigation for newly diagnosed, treated and post-treatment of people living with breast cancer in interior region”. The aim was to co-create a knowledge translation initiative with community stakeholders to enhance interdisciplinary approaches to psychosocial oncology. Methods: This study drew on implementation science and the consolidated framework for implementation research, which emphasize the importance of creating partnerships between researchers and engaging people for whom the research is meant to be of use—knowledge users and service users. Guided world café and purposeful sampling were used to engage a diverse range of stakeholders. Eighty stakeholders participated in this study from April 2023 to April 2024. Thematic analysis was conducted through familiarization, coding, theme development, review, definition, and reporting. Results: Eleven key themes emerged, including compassionate connection, time as a healing gift, empowering health literacy, informed compassion, holistic support ecosystem, empowering patient navigators, shared decision-making, empowering partnerships, digital–physical synergy, person-centered transformation, and accountability and collaboration. Conclusions: The key findings highlighted the need for continuous professional development for primary care providers, integrating patient-reported outcomes in electronic health records, leveraging digital health tools, and establishing community-engaged psychosocial oncology hubs to enhance care in rural communities. Recommendation: Recommendations include ongoing professional learning, embedding patient voices and lived experiences into care planning through digital tools, and empowering rural and diverse communities through inclusive and accessible cancer models of care.

## 1. Introduction

Canada has achieved significant innovations in cancer prevention, screening, diagnosis, and treatment. These innovations demonstrate how evidence-informed knowledge and practices have increased survival rates and enhanced the quality of life for people affected and their caregivers. Cancer incidence is 247,100 cases with 88,100 deaths and a 64% five-year survival rate in Canada, compared to 31,800 cases and 11,700 deaths in British Columbia [1]. The highest cancer incidences in Canada are lung and bronchus (11.6% and 14.4%; 32,100 cases), breast (25.4%; 30,800 cases), prostate (21.9%; 27,900 cases), colorectal (11.1% and 9.3%; 25,200 cases), and bladder (7.3%; 12,300 cases) in males and females, respectively [1]. However, despite medical, technological, and scientific advancements, a critical gap persists in how interdisciplinary approaches to psychosocial oncology address the challenges of creating equity-oriented care in rural and remote communities. Knowledge translation (KT) is a critical process for the consistent implementation of interdisciplinary approaches to psychosocial oncology research in rural community-based interventions. In oncology care, KT is pivotal in ensuring that evidence-based advancements, such as early detection strategies, innovative treatments, and survivorship care models, are implemented effectively. Healthcare practitioners and people living with cancer play an active role in improving reported outcomes [2]. A gap persists in translating these advancements into equitable, psychosocially responsive cancer care in rural and remote communities, where geographic isolation, workforce shortages, and sociocultural barriers continue to limit the reach of supportive care. Although psychosocial oncology has evolved as an essential component of cancer care, its implementation in rural areas has lagged behind due to systemic and contextual barriers.

Dixon-Woods et al. [3] highlighted the challenges of achieving sustained quality improvement in healthcare settings, noting the importance of leadership support, resource allocation, and collaborative structures to foster KT initiatives. The variability in psychosocial oncology models of care across Canada highlights the necessity of locally tailored approaches to KT, such as leveraging telehealth and digital tools to address inequities in rural communities [4]. Building on this, Jull et al. [5] emphasized the importance of addressing systemic inequities in accessing psychosocial oncology services in Indigenous, immigrant, and refugee populations. The lack of culturally sensitive care can hinder trust and engagement with healthcare systems, highlighting the importance of tailored KT approaches that involve community stakeholders in research and decision-making processes. In addition to cultural considerations, rural communities face challenges, such as limited access to specialists, transportation difficulties, and reduced availability of support services, which further complicate the translation of evidence into practice [6]. Previous KT initiatives have focused on urban centers and specialized hospitals, overlooking rural areas where structural inequities persist. Variability in psychosocial oncology models of care across Canadian provinces has the need for tailored KT strategies that adapt to regional realities, such as limited access to specialists and culturally diverse populations.

The community-based participatory research (CBPR) approach complements KT by involving communities in the design, execution, and evaluation of cancer-related interventions. McFarlane et al. demonstrated how CBPR can bridge cultural gaps and address the unique needs of Black, Asian, and other minority ethnic populations, thereby fostering equitable and inclusive KT processes [7]. Cancer survivorship represents complex sociocultural, linguistic, healthcare, and patient–physician relationships that affect quality of life [8]. KT initiatives targeting older adults have identified the importance of addressing age-related disparities, which often stem from systemic ageism and the inadequate integration of geriatric oncology into routine practice [9]. In this regard, educational initiatives targeting healthcare providers have proven effective in enhancing adherence to clinical practice guidelines and improving patient care [10]. Community engagement initiatives, such as KT, supportive care, survivorship care, and navigator programs, promote trust, cultural sensitivity, and collaboration. These initiatives help ensure that interventions are person-centered and contextually relevant in rural communities. Most CBPR projects in psychosocial oncology have focused on education, prevention, and screening programs, with fewer initiatives explicitly linking community engagement to the implementation and sustainability of psychosocial oncology. As a result, rural areas remain underrepresented in KT and CBPR efforts, and their insights are seldom integrated into cancer models of care. Previous KT initiatives have faced challenges in operationalizing interdisciplinary collaboration in rural areas due to siloed funding structures, uneven digital infrastructure, and limited inclusion of patient, caregiver, and care partner voices in decision-making.

Studies [11,12] included a diverse group of healthcare organizations, specialties, and roles that have a part in psychosocial oncology care. Strong partnerships among various stakeholders contribute to the success and effectiveness of the initiative [13]. Coordination encompasses communication, training, educational initiatives, addressing barriers to care, and collaborative efforts among stakeholders. In this context, Tomasone et al. aimed to develop and examine initiatives that cater to the needs of people with lived experiences in cancer survivorship and follow-up care through facilitated discussion between the primary care sector and oncology specialists [13]. The studies identified a lack of effective knowledge dissemination for healthcare providers and highlight that dissemination efforts should account for cultural contexts, community needs, stakeholder engagement, and feasibility. This initiative responds directly to the lack of participatory KT models tailored for rural and community-based psychosocial oncology.

This study reports on a Community-Engaged Knowledge Translation (CEKT) event hosted in British Columbia, built on the project “Enhancing cancer navigation for newly diagnosed, treated and post-treatment of people living with breast cancer in interior region”, which sought to amplify the voices of stakeholders. This study was conceived out of the need to create inclusive community engagement in psychosocial oncology, driven by the CIHR Planning and Dissemination Grant project titled “Co-creating a community engagement knowledge translation world café on improving cancer prevention, screening and posttreatment in Canada”. This study seeks to demonstrate how inclusive dialogue and knowledge co-creation can enhance interdisciplinary coordination, cultural sensitivity, and person-centered care.

### Objective

To co-create a knowledge translation initiative with community stakeholders to enhance interdisciplinary approaches to psychosocial oncology.

## 2. Materials and Methods

### 2.1. Design

This study used Implementation Science and the Consolidated Framework for Implementation Research (CFIR). CFIR includes five domains: outer setting (community needs, funding policies, and advocacy groups), inner setting (technology capability, organizational priorities, and communication networks), intervention characteristics (education materials, patient populations, and health records), process (stakeholder engagement, community partners, and delivery models), and individual characteristics (providers knowledge, patient preferences, and implementation adaptability) to offer a guide for enhancing knowledge translation [14].

### 2.2. Sample Size and Inclusion Criteria

The principles of purposeful sampling were followed. We invited one hundred stakeholders, comprising academic researchers (ARs), healthcare practitioners (HCPs), community service providers (CSPs), lived experiences people (LEP), family caregivers (FCs), and interdisciplinary students (IS).

Inclusion criteria included adults above 18 years, engaging, collaborating, partnering, and caring for people with cancer living in rural communities of Cariboo, Central Okanagan, Columbia-Shuswap, Okanagan-Similkameen, and Thompson-Nicola in the British Columbia Interior. Stakeholders included academic researchers (ARs), healthcare practitioners (HCPs), community service providers (CSPs), lived experience people (LEP), family caregivers (FCs), and interdisciplinary students (IS).

### 2.3. Data Collection Instrument

A community advisory board (CAB) comprising of academic researchers (*n* = 3), healthcare practitioners (*n* = 3), community service providers (*n* = 3), lived experience people (*n* = 2), family caregivers (*n* = 2), and interdisciplinary students (*n* = 2) designed, implemented, and evaluated the Community Engaged Knowledge Translation (CEKT) event in British Columbia. The CAB created and developed the world café, facilitated discussions, and synthesized posters to identify the agenda and prioritize gaps in interdisciplinary approaches to psychosocial oncology. Six main questions, focused on research priorities, were created by the CAB (see Appendix A.1 World Café: Guided Questions).

### 2.4. Data Collection

The world café aimed to facilitate collaborative dialogue, active listening, and roundtable discussion while ensuring accessibility and inclusivity. Each group was guided by trained facilitators using key questions and cues to encourage constructive discussions and the exploration of research priorities. Facilitators were trained by an expert lead guide. Trained facilitators included academic researchers (*n* = 2), community service providers (*n* = 2), lived experiences people (*n* = 2), and interdisciplinary students (*n* = 4). Collaborative team-building sessions were interspersed with panel discussions to synthesize insights and strategies. Key discussions and decisions were captured through notetaking, audio recordings, and poster summaries. To begin the discussion, the table and online facilitators read aloud the question written on the flip chart and informed the participants that they may hand in written points at the end if time does not allow the point(s) to be included (see Appendix A.2 World Café Facilitator Guidelines). The facilitators then invited the participants to share their experiences and perspectives; the total time was six hours. After the discussion with the entire group, the participants moved to a new table on the other side of the room, sitting with as many new participants as possible. Online participants may switch rooms, as determined by the online facilitators. The keynote facilitator again invited each facilitator to read the discussion points from their tables and the online group’s questions. The participants then responded to the same questions as in the first round during a general group discussion. The graphic illustrator made visual representations of the discussion points, themes, subthemes, general comments, and responses to the questions. After the group discussions, the key points and suggestions brought forth by the participants were collated, analyzed, and synthesized into posters, serving as visual representations of the insights contributed by the world café participants. The report summarized discussions highlighting innovative suggestions to foster equitable, collaborative, and contextually responsive care. This study was conducted from April 2023 to April 2024.

### 2.5. Ethical Considerations

Ethical approval certification was obtained. All participants provided informed consent, and their confidentiality and anonymity were maintained. They were provided with gift cards as tokens of appreciation for their contributions.

### 2.6. Data Analysis

Following Braun and Clarke’s framework, thematic analysis was conducted using a systematic and reflective process, including familiarization, coding, theme development, review, definition, and reporting [15]. Steps followed were: (1) We immersed ourselves in the transcripts by reading and re-reading them to gain a deep understanding of the content. During this phase, we noted initial impressions, emerging ideas, and patterns while maintaining reflective memos to capture early analytical insights and contextual observations. (2) Using NVivo 14 software, we systematically coded the data by identifying meaningful and recurring features. Each segment was coded comprehensively to ensure inclusiveness. Reflexive journaling was used to document analytical decisions and maintain transparency throughout the coding process. (3) We organized related codes into broader, conceptually meaningful patterns aligned with the research objectives. Thematic maps were developed to visualize relationships among codes and to guide the clustering of potential themes through iterative interpretive analysis. (4) All preliminary themes were refined for internal coherence and external distinction. We revisited the coded data extracts to ensure consistency and accuracy, merging overlapping themes and redefining boundaries where necessary. Updated thematic maps were generated to reflect the refined structure. (5) Each theme was clearly defined to capture its core meaning and relevance to the research aims. Concise and reflective names were assigned, supported by detailed descriptions and illustrative participant quotes to enhance interpretive clarity. (6) The final phase involved weaving the themes into a coherent analytical narrative. The findings were presented with supporting data extracts and interpretive commentary, linking insights to existing literature.

## 3. Results

We had a response rate of 80%. Stakeholders comprised of academic researchers (AR) from nursing (5), business (2), science (2), economics (1), education (1), and social work (1) programs (*n* = 12); healthcare practitioners (HCP), including family physicians (4), registered nurses (5), physiotherapists (2), dieticians (2), radiation therapists (1), and pharmacists (1) (*n* = 15); community service providers (CSP) (*n* = 16), including social workers (5), massage therapists (4), counsellors (4), and care coordinators (3); lived experience people (LEP) at various stages of cancer diagnosis (3), treatment (3), and survivorship (5) (*n* = 11); family caregivers (FC) *(n* = 12); and interdisciplinary students (IS) (*n* = 14) from nursing (5), business (3), science (2), economics (2), education (1), and social work (1) programs.

### Thematic Analysis

The community engagement knowledge translation (CEKT) framework included establishing accountability, fostering knowledge discovery, and collective learning (Figure S1). The eleven themes from the world café were compassionate connection, time as a healing gift, empowering health literacy, informed compassion, holistic support ecosytem, empowering patient navigators, shared decision-making, empowering partnerships, digital–physical synergy, person-centered transformation and accountability and collaboration. These themes support meaningful community connections and interdisciplinary partnerships among stakeholders and lay the foundation for psychosocial oncology research and practice (Figure S2).

**Category** **1.** *Facilitating open conversations with patients living with cancer and their caregivers.*

Two emerging key themes were Theme 1. Compassionate connection and Theme 2. Time as a healing gift.

*Theme 1. Compassionate connection*

Participants described communication as a dynamic process shaped by empathy, trust, and structural factors such as time and resources. Their narratives illustrate the mechanisms of implementation science, such as relational integration and sense-making processes, where effective interactions depend on emotional attunement, clarity, and mutual understanding.

One participant replied, “Building trusting relationships, such as cultivating rapport over time, allowing patients to confide and share openly. Developing a positive image and approachability, such as presencing a warm, approachable demeanor that encourages trusting patient interaction. Demonstrating genuine interest, such as showing sincere concern for patients’ well-being, beyond clinical assessments, is important.” LEP#2.

Participants highlighted that relational trust stems from authentic, face-to-face engagement and the effective use of nonverbal communication. One participant described “building trusting relationships” and “allowing patients to confide and share openly,” illustrating relational coherence, a determinant of successful implementation that develops through sustained presence, emotional resonance, and active listening. Their emphasis on a positive demeanor, eye contact, and approachable language reflects the operationalization of relational coordination, which links empathy and shared goals to improved communication flow.

Another participant stressed, “Face-to-face interaction, like fostering genuine, empathetic connections with patients through direct, personal engagement. Paying attention to personal body language, using plain informal language, communicating in a relatable manner and avoiding medical jargon helps to ensure patients feel heard and understood.” LEP#9.

Encouraging practitioners to minimize jargon and adopt relatable, plain language, participants aligns with the recommendations with CFIR’s construct of intervention adaptability. This adaptability allows practitioners to tailor communication strategies to patient readiness, thereby promoting patient-centered care as a normalized practice rather than an aspirational value.

*Theme 2. Time as a healing gift*

Participants consistently described time not only as a logistical factor but also as a therapeutic resource. One social worker stated that “adequate interaction time” allows patients to “feel heard,” illustrating how temporal structures shape relational quality. This aligns with implementation climate constructs, in which time allocation signals the organization’s prioritization of person-centered interactions.

With one participant stating, “Adequate interaction time, such as ensuring healthcare providers have sufficient time to engage and interact with patients. XX cancer social worker support recognizes the critical role of social workers in providing time, resources, and emotional support. The gift of time in open conversations allows patients to feel heard during conversations with their provider.” LEP#72.

Calls for the interoperability of health data, collaborative record transfer, and streamlined data-sharing represent systemic enablers within CFIR’s inner setting domain, supporting coordination between disciplines and ensuring continuity of care. Participants’ references to digital tools-assisted documentation resonate with the concept of technological facilitation in implementation frameworks, enabling providers to prioritize relationship-building over administrative burdens.

Another participant voiced the need for “Collaborative timely record transfer, such as exploring efficient ways to transfer patient records across authorities for seamless follow-up care. A common patient database, like creating a shared repository for easy access to patient information during initial conversations with different physicians, is helpful for continuity and follow-up.” FC#21.

The participants also discussed the importance of timely collaboration among authorities to ensure the efficient transfer of patient records. This enhances follow-up care, improves continuity, and facilitates initial conversations with different physicians. Healthcare communication often involves vocabulary that is unfamiliar to patients and requires additional explanation to ensure patient comprehension of cancer-related terminology.

Use of reframing time as an enabler of empathy and coordination, participants linked micro-level communication practices to macro-level implementation determinants, highlighting that relational quality thrives within supportive systems that value continuity and human presence.

One participant stated, “Assessing patients’ level of health literacy to comprehend and communicate health information to promote one’s health and readiness or preparedness to adopt and implement new behaviours for providing the right information at the right time in the right way. Peer support and online resources need to be evidence-based, user-friendly, accurate and available to connect with. Artificial intelligence assisted documentation using technology to record and transcribe information will allow practitioners to focus fully on patients during consultations.” HCP#33.

**Category** **2.** *Psychosocial challenges associated with cancer treatment and posttreatment.*

Two key themes that emerged were Theme 3. Empowering health literacy and Theme 4. Informed compassion.

Participants recognized psychosocial adaptation during and after treatment as a process requiring both informational empowerment and emotional support. Their accounts mapped onto the constructs of capacity building and readiness for change within implementation science, emphasizing how interventions must align with individual literacy levels, motivation, and emotional readiness.

Another participant responded, “Using senior navigators to help connect with available support and use self-questionnaire to address needs, barriers and resources. What do you need to thrive? How would you like to talk? How can you be better? And we want this.” CSP#45.

*Theme 3. Empowering health literacy*

One participant highlighted, “Assessing knowledge levels like understanding patients’ existing understanding of cancer and related issues. Health literacy readiness, like providing information at the right time and in a way that matches patients’ readiness to absorb it. Active listening is using the 18-s rule to truly hear patients’ concerns and tailor responses accordingly. We need more time to transition and decompress.” CSP#46.

Participants advocated for the ongoing assessment of patients’ knowledge and health literacy readiness, stressing that information delivery must match individual learning needs and timing. This reflects the individual-level determinants within CFIR, particularly knowledge and beliefs about the intervention. One participant described using the 18-s rule to actively listen and tailor communication, an example of reflexive monitoring in the normalization process, where practitioners continually adapt interventions based on patient feedback.

Another participant mentioned, “Evidence-based online resources help to guide patients to reliable, accurate online materials. Having peer support groups for senior care and increasing peer support networks for meeting emotional and informational assistance in the community. Establish a central hub or society with specific programs, supports and resources to serve the people diagnosed with cancer and their caregivers.” FC#28.

Peer community networks and evidence-based online tools were cited as critical resources for knowledge democratization. These approaches embody the principles of diffusion of innovation, wherein credible information sources and modeling by peers enhance the adoption and sustainment of self-management behaviors across the cancer care continuum.

*Theme 4. Informed compassion*

Participants emphasized the need for authentic conversations beyond clinical data, capturing the essence of congruence between cognitive and relational work in implementation processes. The integration of digital tools and structured documentation, such as SOAP or SOAPIER notes, aligns with processual constructs, enhancing accountability, documentation accuracy, and communication across teams. However, this mechanistic precision was perceived as meaningful only when paired with compassion and contextual sensitivity.

One participant mentioned, “Having patient-centric assistance helps to identify and address gaps in patients’ knowledge. Having technology and AI leveraging tools enhances communication, record-keeping, and translation. Engaging in real conversations with patients in meaningful dialogues goes beyond clinical data. Reviewing Subjective, Objective, Assessment and Plan (SOAP) notes, using concise, structured notes to communicate effectively within the healthcare team helps to ensure continuity by revisiting previous interactions.” HCP#74.

Informed compassion thus represents an implementation mechanism that transforms standardized processes into personalized care experiences, sustaining trust through continuity, follow-up, and a sense of being truly seen and remembered across encounters.

**Category** **3.** *Innovative strategies to empower patients and caregivers in actively participating in decision-making processes.*

Two emerging key themes were Theme 5. Holistic support ecosystem and Theme 6. Empowering patient navigators.

Participants’ descriptions reflected co-production principles in implementation science, positioning patients and caregivers as active agents in designing and enacting care processes. Their insights link to outer setting constructs within CFIR, emphasizing community partnerships, accessible resources, and equitable system navigation.

One participant stated, “Recognizing patients as experts in their care and providing evidence-based information on available alternatives provides an opportunity to take an active role in maintaining their goals and decision support tools for treatment choices. Engaging patients to become autonomous allows them to express their goals and needs for ensuring the care they need.” HCP#37.

*Theme 5. Holistic social support networks*

Participants called for integrating community-based peer programs, caregiver respite opportunities, and psychosocial support systems. Their accounts reflected relational coordination mechanisms, where trust, respect, and shared understanding among patients, families, and practitioners lead to adaptive teamwork. The frequent reference to questions such as “What do you need to thrive?” features a co-design orientation, a process that operationalizes learning health system principles by incorporating patient feedback into service design.

One participant stated, “Peer engagement encourages patients to connect with others who share similar experiences in cancer care. Caregiver time helps to allocate sufficient time for caregivers to actively participate in patient care. Transition and decompression allow patients and caregivers space to process emotional shifts during treatment and post-treatment phases. Individualized support helps to tailor assistance to each patient’s unique psychosocial needs.” CSP#1.

Another participant stated, “Communication is creating open channels for dialogue among patients, caregivers, and healthcare providers and helps to address uncertainty and side effects and ensures emotional support and practical guidance. Effective treatment collaboration will foster teamwork among healthcare providers, patients, and caregivers and the use of assessment tools such as questionnaires to identify barriers, needs, and required resources.” AR#50.

Another participant indicated, “Financial and emotional considerations acknowledge and address the emotional and financial burdens of patients. Professional navigators who have access to resources will assist them to better connect patients with available support and services and ensure a person-centered approach to base interactions. Equipping patients to make informed decisions and allowing them to make their own choices empower[s] patients and helps to build partnerships.” FC#80.

The integrated model of peer engagement, communication transparency, and caregiver inclusion reflects system-level organizational readiness, promoting sustainability and emotional resilience throughout the cancer care continuum.

*Theme 6. Empowering patient navigators*

Participants emphasized the role of navigators as relational intermediaries connecting patients to resources, reducing fragmentation, and enhancing trust. This aligns with constructs of implementation facilitation, where specific roles enable coordination, problem-solving, and patient empowerment. Timely connections, feedback loops, and continuous learning illustrate iterative cycles of reflection and adaptation embedded in effective implementation.

One participant stated, “We need a range of support from seniors to all age groups, empower patient navigators to guide patients. We need timely connections to promptly link patients to relevant services and peer networks. We need continuous learning, adaptation and staying open to feedback and learning from patient experiences. We need to provide psychosocial support and allocate time for emotional transitions and taking care of side effects. We need to initiate a community hub for seeking supportive care and survivorship resources.” AR#59.

**Category** **4.** *Creating a supportive environment that encourages ongoing communication.*

Two emerging key themes were Theme 7. Shared decision-making and Theme 8. Empowering partnerships.

Recognizing and addressing silos, facilitating better information sharing, and developing partnerships across private and public sectors fosters a supportive environment and shared responsibility in psychosocial oncology. The focus is on developing supportive care and survivorship that facilitates shared responsibility among patients, caregivers, and care partners that is responsive to the needs of patients. Personalized definitions of care, clear expectations, and established accountability are necessary to create a supportive environment between patients and healthcare providers, with distinct roles and responsibilities.

*Theme 7. Shared decision-making*

One participant stated, “Having informed choices involves patients and caregivers in treatment decisions by providing clear information on the risk-benefits, potential side effects, trade-offs, and quality of life implications. Setting collaborative goals for realistic treatment goals is aligned with patients’ values and priorities.” AR#76.

Another participant stated, “Health literacy enhancement helps to ensure patients understand treatment options and their implications. Caregiver inclusion helps to acknowledge caregivers’ roles in decision-making and consider their perspectives. There is a need for clarity, domain expertise, and a person centered approach to promote effective communication. Oncology physiotherapist roles, scope, patient profile, regulatory guidelines, and collaboration are critical to focus on to provide a clear understanding of the interdisciplinary team.” IS#61.

One participant highlighted, “Separating family expectations from clinical realities to aid the emotional strain of caregivers and families of cancer patients. Defining roles, objectives for setting priorities and agendas that may not be fully aligned to create supportive and survivorship care to dismantle the impact of silo systems.” AR#53.

Hence, incorporating strategies that promote informed choices and engage patients in risk–benefit discussions, can help healthcare teams ensure that treatment decisions align with patient values and priorities. The necessity of collaborative goal setting and enhancing health literacy enables patients to fully understand their treatment options and their implications. There is a gap in addressing the transition, continuity, and coordination of care for patients and a lack of reality and expectations of caregivers and patient support systems.

*Theme 8. Empowering partnerships*

Patients should be acknowledged as experts in their health. Critical insights help to understand patients’ stories and voices to forge meaningful connections and recognize each patient’s path through cancer care. Advocating for increasing autonomy and choice allows patients to actively participate in informed decision-making and informed choices.

One participant mentioned, “Community capacity building provides timely, accurate information tailored to community needs and openness to partnerships and collaboration with diverse stakeholders for comprehensive care. Transparent team dynamic effects foster fluidity and trust from the outset, prevent burnout and prioritize team well-being to sustain effective care. Evidence-based alternatives equip patients with reliable treatment information.” IS#65.

**Category** **5.** *Digital tools be utilized to foster continuous communication and collaboration.*

One emerging key theme was Theme 9. Digital–physical synergy.

Integrating digital tools into continuity care with consent, confidentiality, and comfort is considered important to improve care coordination. This includes addressing privacy concerns, easing patient interactions with technology, and developing universal record systems to streamline care and support healthcare experiences. The continuous development of information, communication, education, and technology creates innovations within healthcare with psychosocial oncology benefits.

*Theme 9. Digital-physical synergy*

One participant emphasized, “Personalized options to blend digital and in-person approaches based on patient preferences and offering resources aligned with patients’ chosen modes of interaction. Building trusting relationships through transparent communication and learn from lived experiences, stories, voices, and case studies.” IS#68.

Another participant highlighted, “Street nurses explore innovative outreach programs, including street nursing for people experiencing homelessness and treated with cancer. Empowering partnerships and strengthening collaborations through capacity building is valuable to disseminate accurate information effectively to create a supportive environment for patients. Fluid team dynamics helps to adapt to changing needs and challenges.” CSP#38.

In bridging the digital and physical aspects of healthcare, there is a need to incorporate digital and in-person options based on patient preferences, ensuring that care delivery is adaptable and person-focused. The support for this hybrid approach was extended by providing support for chosen preferences. Strategies such as the use of street nurses for outreach programs were considered vital in reaching wider communities, enhancing the accessibility and responsiveness of psychosocial oncology.

**Category** **6.** *Innovative strategies and ways to ensure collaborative and supportive cancer care.*

Two emerging key themes were Theme 10. Person-centered transformation and Theme 11. Accountability and collaboration.

The creation of information platforms and clear communication programs should ensure equitable access to healthcare. The development of a cancer supportive and survivorship center is essential to foster an inclusive environment for diverse populations living in rural and remote communities. There is a clear need for the identification of psychosocial oncology services and having coordinated information across online resources. Ensuring that psychosocial oncological approaches are promoted to patients will allow for increased accessibility and improved care with adequate resources available in rural communities.

*Theme 10. Person-centered transformation*

One participant stated, “XX Cancer is collaborating with XXX to create a communication program for patients. Decision-making triad of patient choice, knowledge, and expert guidance with digital versus in-person interactions to balance mobile and street nursing. Building strong partnerships help to empower patients, caregivers, and healthcare providers. Capacity building helps to disseminate accurate information effectively while creating trust and communication to foster trusting relationships.” CSP#35.

The development of an accessible communication model for patients across different health regions is valuable. Participants brought focus to the need to obtain informed consent from patients before community initiatives, such as collaborations, and to provide clear information. The advocacy of stakeholders works to achieve health equity and provide care for communities in desperate need of local access to care and programs for psychosocial oncology.

*Theme 11. Accountability and collaboration*

The significance of shared responsibility, including accountability, reflection, and awareness of what you can and cannot do in managing care, was emphasized. Stakeholder roles were further clarified through identifying areas of responsibility, shared duties, and the collaborative engagement, fostering a unified approach to care. Enhancing education through the oncology physiotherapy role, scope, patient profiles, regulatory considerations, and collaboration emphasized the need for continuous learning and role clarification to optimize patient outcomes. The importance of synthesizing available knowledge to provide overlap and reduce friction or barriers highlights the necessity of communication within psychosocial oncology.

One participant stated, “Shared responsibility with reflection on roles and limitations. Creating an agenda to tie accountability to defined care objectives and shared areas to clarify responsibilities across stakeholders. Sustaining local community programs like supportive care, survivorship, education and support. Managing family expectations to reduce stress.” LEP#54.

Another participant stated, “Evidence-informed care based on decisions on patient needs, physician attribution to acknowledge collective accountability to create a person centered lens and collaboration from the patient’s perspective. Psychosocial oncology rehabilitation referrals to address gaps in continuity of care. Creating an effective referral system to streamline patient access to specialized care, education and role definition to enhance person-centered care.” HCP#23.

The conceptual integration of outreach nursing, psychosocial support, and community hubs reflects a multi-level systems approach in rural communities. It demonstrates how implementation science combined with structural supports, transparency, and shared decision-making processes can normalize patient engagement and empowerment as routine evidence-informed practice within community oncology networks.

## 4. Discussion

This study sought to co-create a knowledge translation (KT) initiative with community stakeholders to enhance interdisciplinary approaches to psychosocial oncology. Applying the CEKT framework, this research demonstrated how participatory knowledge exchange can bridge the gap between research evidence, professional practice, and community experience in rural communities. The findings extend existing implementation science frameworks by illustrating how relational, cultural, and contextual factors can be operationalized to foster sustainable, person-centered psychosocial oncology practices. Within the CFIR, these findings align with outer settings (community needs, funding policies, and advocacy groups), inner settings (technology capability, organizational priorities, and communication networks), intervention characteristics (education materials, patient populations, and health records), processes (stakeholder engagement, community partners, and delivery models), and individual characteristics (providers knowledge, patient preferences, and implementation adaptability).

This study revealed that psychosocial oncology interventions are more successfully implemented when healthcare providers communicate in ways that humanize care and acknowledge emotional realities, thus increasing patient trust and engagement. This finding resonates with the role of effective communication and information in cancer care [16,17]. Barry and Edgman-Levitan stressed the need to engage with patients’ emotional and nonverbal cues to fully grasp their concerns and fears, which can affect their care and reported outcomes [18]. Health literacy enhances patient engagement in care by helping them make informed decisions and engage actively in their care [11]. The demeanor and appearance of healthcare providers can set the tone for open communication, encouraging patients to share emotional, financial, and health concerns freely [19]. In rural communities, where continuity of care is challenged by distance, limited resources, and social isolation, compassionate communication builds relational infrastructure that supports the sustained uptake of psychosocial innovations. Providing a warm and approachable demeanor creates a safe environment for patients to interact freely and openly with the provider [20]. Avoiding the use of medical terms was also mentioned to ensure that patients felt understood and heard in conversations focused on complex medical concerns or therapies [21]. This finding deepens KT theory by framing empathy as a translational process, transforming evidence-based psychosocial principles into lived relational practice that strengthens therapeutic alliances.

Empowering health literacy emerged as a pivotal strategy for ensuring that evidence-based cancer information is accessible and actionable. Drawing on the CFIR, this finding emphasizes that successful implementation depends on the interaction between context, evidence, and facilitation. Participants identified culturally responsive, linguistically inclusive, and digitally supported communication as critical facilitators of patient empowerment and shared decision-making. Creating culturally inclusive care in which patients feel genuinely heard improves their engagement and satisfaction with the care process [11]. Furthermore, leveraging digital tools and guiding patients toward evidence-based online resources can supplement the health literacy efforts made during clinical encounters [22]. Similarly, using plain, informal language helps empower patient health literacy by tailoring discussions to each individual’s readiness to learn [23]. Other studies have shown that fostering genuine relationships between healthcare providers and patients builds a trusting relationship and is the most effective way to encourage open conversations [24]. This insight contributes to KT practice by extending the traditional notion of dissemination to include adaptive communication, tailoring information delivery based on literacy level, cultural identity, and readiness to engage. Providing a warm and approachable demeanor creates a safe environment for patients to interact freely and openly with the provider [20]. Avoiding the use of medical terms was also mentioned to ensure that patients felt understood and heard in conversations focused on complex medical concerns or therapies [21]. In rural communities, digital literacy initiatives and trusted online resources can complement in-person care and bridge geographical barriers while enhancing psychosocial support continuity. Thus, health literacy functions as both an outcome and a driver of implementation success.

The finding of time as a healing gift highlights temporal constraints as structural determinants of psychosocial oncology implementation. Participants described how limited clinical time hinders empathetic dialogue and co-created care planning. Within implementation science, this theme intersects with the CFIR construct of available resources and collective action. Allocating sufficient time allows healthcare providers to engage meaningfully with patients, enact compassionate behaviors, and embed psychosocial care practices within routine workflows. Studies have emphasized that allocating sufficient time for patient interactions strengthens trust, supports meaningful engagement, and improves communication and satisfaction with care [20]. Gupta et al. reported shifts in healthcare practice, showing that time allocation influences patient outcomes [25]. Adequate time allocation allows healthcare providers to demonstrate their commitment to patients’ well-being. Nardone et al. discussed the transformative role of digital communication in cancer care, enhancing decision-making processes and optimizing treatment plans [22]. In rural communities, innovative scheduling models, team-based task sharing, and the integration of digital tools for administrative tasks can free provider time for psychosocial engagement, transforming “time” into an enabler of implementation fidelity and patient-centered care.

The theme “informed compassion” expands on the interplay between empathy and structured knowledge sharing within interdisciplinary teams. Participants emphasized that compassion must be supported by accurate, shared, and timely information to maintain continuity of psychosocial care. This reflects principles from the CFIR framework, particularly the phases of adapting knowledge to context and monitoring knowledge use. Structured communication tools and team debriefing practices enhance relational coordination and ensure that psychosocial knowledge is consistently translated across professional boundaries. Through educational interventions, healthcare providers can ensure that patients understand the implications of their treatment choices, thus empowering them to make informed decisions [2]. The integration of digital tools into healthcare can personalize communication and educational materials based on literacy, language, and cultural needs, enhancing patient engagement, understanding, and empowerment [22]. Clarity in communication and the recognition of cultural differences helps to improve reported outcomes and enhance the quality of life of people with cancer [17]. The use of structured communication tools within healthcare teams was advocated to enhance the effectiveness of informed compassion and to ensure that all team members are consistently aware of the patient’s condition, treatment plans, and any psychosocial factors that may affect their care [26]. This approach maintains high standards of care and ensures that compassion is informed by accurate and updated patient information [27]. This finding advances KT theory by proposing informed compassion as a form of team-level knowledge translation, where emotional intelligence and contextual evidence coalesce to sustain interdisciplinary learning cycles. In rural communities, this approach mitigates fragmentation and fosters a unified psychosocial care identity across healthcare practitioners and community care providers.

The identification of patient navigators as central to equitable psychosocial oncology reinforces their role as knowledge makers within implementation systems. From an implementation science perspective, navigators function as facilitators who mediate between system-level policies and patient-level experiences, thereby operationalizing KT through individualized support and coordination. Empowering professional navigators emerged as a pivotal strategy for enhancing cancer care delivery. The participants highlighted patient navigators as essential liaisons who provide timely, tailored support, bridging patients and the healthcare system [28]. Such tailored support is crucial in building resilience and social capital, which are significant for successful psychosocial oncology approaches [29]. Centralizing resources in the community helps people living with cancer connect more efficiently with the necessary services and improves social support networks [24]. At the policy level, new cancer action plans created by healthcare policymakers and healthcare practitioners aim to address the needs of people in rural communities [2]. Using personal assessment tools allows for a patient-driven approach that empowers patients to engage in their care and identify their needs [30]. In rural settings, expanding navigator roles to incorporate cultural competency, self-assessment tools, and digital engagement platforms provides a cancer model of care for integrating psychosocial care across dispersed communities. This aligns with the CFIR, illustrating how navigator-driven initiatives can enhance reach and sustainability by ensuring that psychosocial interventions remain contextually responsive and equity-focused.

The emphasis on holistic support networks reflects an ecosystemic understanding of psychosocial oncology, where care extends beyond clinical boundaries into community spaces. These social support and oncology networks exemplify implementation science, positioning local leaders, caregivers, and peer groups as co-implementers of psychosocial interventions. In this study, where Indigenous, Black, Asian, and Minority Ethnic populations face systemic access barriers, culturally grounded peer networks, and community oncology hubs create relational continuity, reduce isolation, and facilitate the localized adaptation of KT initiatives. This finding enriches KT theory by demonstrating that contextual co-production, the co-design of implementation strategies by those embedded within the setting, is essential for achieving sustained, equitable outcomes. Effective communication fosters trust and ensures alignment with treatment goals and expectations, which is fundamental in any therapeutic relationship [31]. Caregivers require adequate support to reduce their burdens and improve care quality [24]. Holistic support networks are essential for continuous communication among patients, caregivers, and healthcare providers, ensuring that both medical and psychosocial needs are addressed.

This study contributes to implementation science by illustrating how Implementation science, CFIR and KT methods operationalize theory into rural oncology practice. The co-creation process anchored in the CEKT model demonstrates that effective KT is relational, iterative, and adaptive, driven by empathy, communication, and shared decision-making rather than unidirectional evidence transfer. The integration of world café dialogues provided a living laboratory for testing how evidence and experience intersect to produce actionable psychosocial care innovations. For psychosocial oncology, this study contributes new knowledge by reframing KT as an interpersonal and cultural process. It highlights how relational empathy, communication literacy, and interdisciplinary trust serve as active ingredients in implementing equitable psychosocial care in resource-limited rural communities.

### Limitations

Although adapting the world café method successfully fostered collective dialogue and priority setting, further follow-up is required to ensure the sustainability and application of the insights generated. The use of a small, purposeful sample drawn from established academic, research, community, and healthcare partnerships limits generalizability. Participants were affiliated with organizations engaged in oncology initiatives, which may have shaped the perspectives represented. The rural community context limits transferability to urban areas, where community structures and health service systems, programs, services, and resources differ. Continued engagement with community and healthcare partners is essential to translate co-created priorities into actionable initiatives and to further advance community oncology networks among knowledge users, researchers, service providers, and people.

## 5. Conclusions

The findings demonstrate that community co-creation and development can serve as a powerful mechanism for translating evidence into culturally responsive, contextually relevant cancer care practices. Using implementation science, this study operationalized KT principles within real-world settings, transforming passive dissemination into active collaboration. This approach enhances implementation fidelity and adaptability in rural communities. KT outcomes include continuous education and psychosocial competency-building for primary care providers, the integration of patient-reported outcomes to guide psychosocial interventions, the use of digital health tools for remote psychosocial support and monitoring, the development of community-led frameworks and caregiver training to reinforce person-centred cancer care, and the establishment of partnerships to enable sustainable, data-informed decision-making.

This study contributes to KT theory by demonstrating how implementation science methodologies can operationalize the CEKT model to generate contextually rich, actionable knowledge. It advances KT practice by integrating reflective dialogue, collective sense-making, and co-implementation planning within an interdisciplinary psychosocial oncology framework. This participatory approach bridges the persistent gap between research evidence and frontline practice by embedding end-users and communities directly into the process of knowledge mobilization. This research repositions KT as a cyclical, community-anchored, and culturally responsive process compared to a linear, top-down model. It offers a replicable CEKT framework for embedding KT processes within local cancer systems to foster mutual learning, system integration, and sustained psychosocial innovation. The innovation lies in positioning psychosocial oncology as a site of CEKT practice, where scientific evidence, lived experience, and social context converge to shape adaptive, equitable care models. The use of the world café as an interactive KT facilitates knowledge sharing and cultivates relational trust, interdisciplinary cohesion, and a shared ownership of implementation goals. This redefines how psychosocial oncology can move beyond clinical silos toward community-informed, system-level resilience and integration in cancer care.

The co-creation approach ensures cultural adaptability by embedding linguistic inclusivity, community leadership, and reciprocal learning and transfer across Canadian provinces and among Indigenous, Black, Asian, and Minority Ethnic communities into KT design. Future applications can leverage this CEKT framework to develop specific KT interventions that address systemic inequities in cancer screening and psychosocial care. The participatory, multi-lingual, and equity-oriented processes identified provide a practical roadmap for embedding cultural safety, local relevance, and community governance into KT and implementation science efforts.

Establishing a continuous professional development program that equips rural primary care and allied health professionals with current knowledge in psychosocial oncology, survivorship care, and culturally responsive practice is essential. The Ministry of Health and health authorities can embed this initiative into existing continuing education frameworks, ensuring sustained funding and alignment with provincial cancer care standards, outcomes, and performance indicators. Developing integrated digital systems that embed patient feedback and psychosocial data directly into electronic health records is a priority. Regional authorities and Cancer Care can operationalize this through collaborative digital infrastructure investments, ensuring that psychosocial data informs both individual care and system-level quality improvement across rural community oncology networks. Strengthening psychosocial oncology support through community-based models of care that prioritize inclusion, cultural safety, and accessibility is essential. Provincial planning leaders and policymakers can reduce access inequities, enhance early intervention, and foster resilience in rural and remote communities through integrating psychosocial services within community infrastructure and fostering partnerships across sectors. These recommendations emphasize the importance of building psychosocial oncology capacity through ongoing professional learning, embedding patient voices and lived experiences into care planning and empowering rural and remote communities through inclusive and accessible equitable cancer models of care.

## Data Availability

The data presented in this study are available on request from the corresponding author due to privacy, legal, and ethical restrictions.

## Supplementary Materials

The following supporting information can be downloaded at: https://www.mdpi.com/article/10.3390/ijerph22121789/s1, Figure S1: Knowledge translation framework; Figure S2: Thematic map.

## Appendix A

### Appendix A.1. World Café: Guided Questions

1. How can healthcare practitioners facilitate open and effective communication between patients living with cancer, their caregivers, and themselves to ensure a complete understanding of the patient’s cancer illness and treatment plan?
2. In what ways can patients, caregivers, and healthcare practitioners collaboratively address the psychosocial challenges associated with cancer treatment and post-treatment, and how can this collaboration enhance the well-being of the patient?
3. What innovative strategies can healthcare practitioners employ to empower patients and caregivers to actively participate in decision-making processes related to cancer treatment and post-treatment, and how does this contribute to building a stronger partnership between all stakeholders?
4. How can healthcare organizations create a supportive environment that encourages ongoing communication between patients, caregivers, and healthcare practitioners, fostering a sense of shared responsibility in the cancer care journey?
5. Considering the evolving information communication technology, how can digital tools be utilized to foster continuous communication and collaboration among patients, caregivers, and healthcare practitioners in the cancer care continuum?
6. What are some innovative strategies and ways we can ensure collaborative cancer care supportive services across different ages, genders, cultural, socioeconomic, and rural communities for people living with cancer and their caregivers?

### Appendix A.2. World Café Facilitator Guidelines

1. The key facilitator guides world café roundtable discussions and gives a knowledge summary and synthesis during the entire process.
2. High-quality personnel as facilitators and notetakers are present at each table in-person and on online forums. Trained facilitators will guide, generate dialogues, give cues, build on the discussions, present and synthesize findings.
3. Participants engage through knowledge sharing and knowledge exchange at the small roundtable and online discussions culminating into large group discussions.
4. Participants in small group discussions create strategic imperatives, innovative solutions and addressing policies for each question, timeframe is 30 minutes. Six-seven participants will be assigned at each table and will switch tables for each question.
5. Large group deliberations and knowledge exchange and provide suggestions to generate rich discussions and synthesis for each question; timeframe is 30 minutes.
6. After small group discussion concludes, invite participants to move quickly to a new table for the next question, with as many different participants as possible.
7. The table and online facilitators make detailed field notes and synthesis on the poster. At the end, each printed poster synthesis are shared in-person and digital images are shared online for knowledge synthesis.
