# Supplementary figures and images for: Knowledge Translation Initiative to Improve Interdisciplinary Approaches to Psychosocial Oncology Among Community Stakeholders in Rural Regions of British Columbia
Source: Int J Environ Res Public Health. 2025 Nov 26;22(12):1789. doi: 10.3390/ijerph22121789 (PMC12732520; doi:10.3390/ijerph22121789)

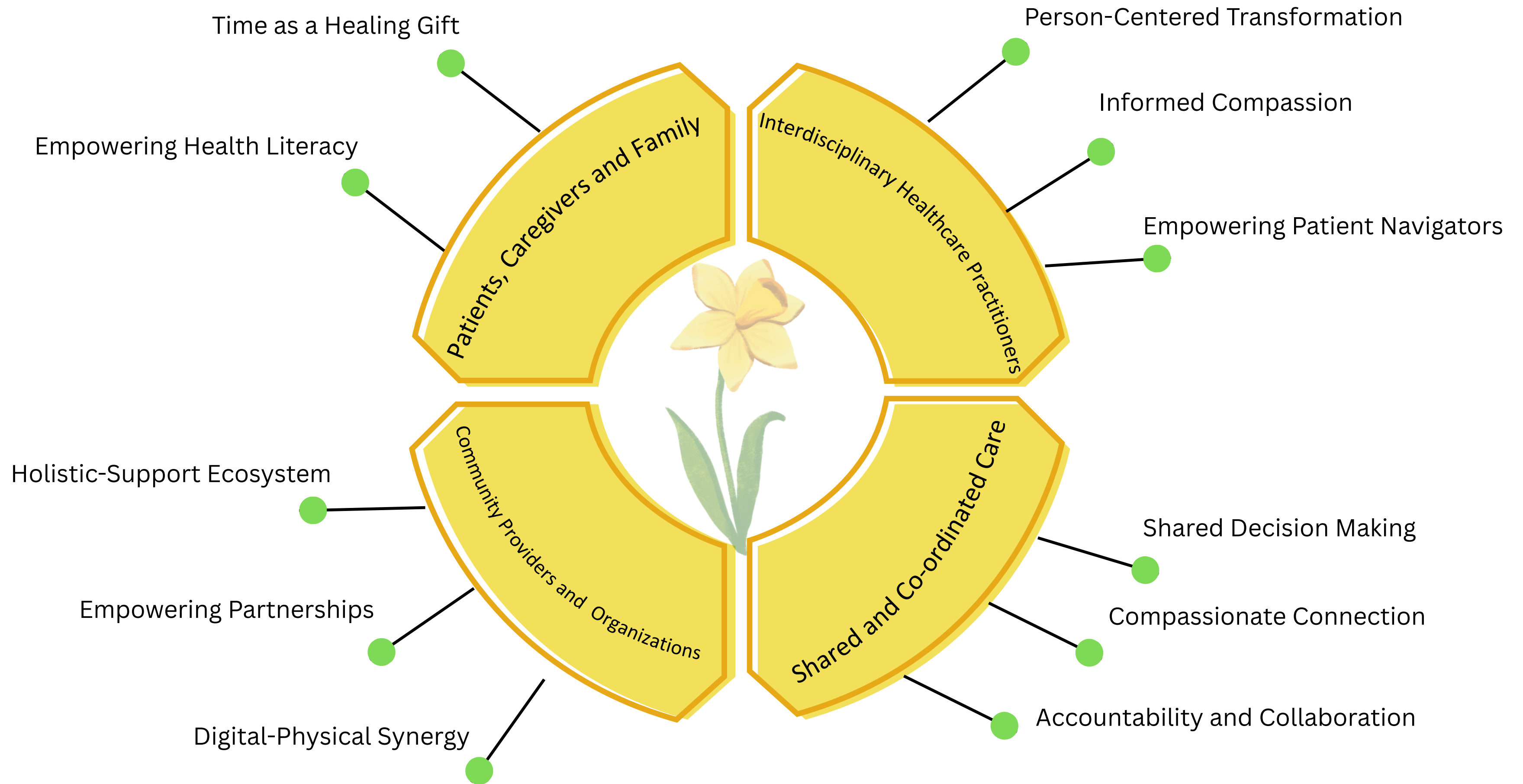

Figure S1. Knowledge Translation Framework

Supplement: Supplementary file 1 [file ijerph-22-01789-s001.zip › ijerph-3939642 - Figure S1.pdf]

Figure S2. Thematic Map

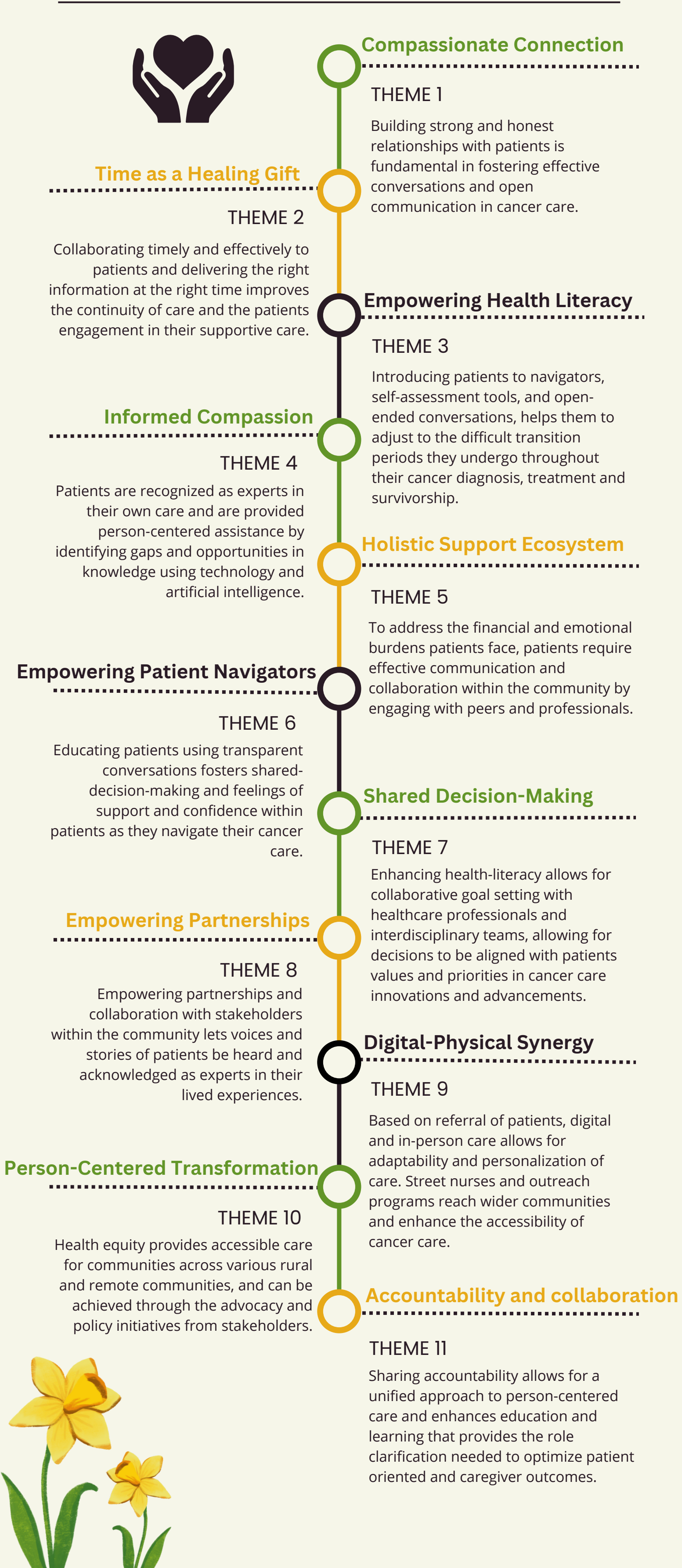

Supplement: Supplementary file 1 [file ijerph-22-01789-s001.zip › ijerph-3939642 - Figure S2.pdf]
